# The Impact of Climate Change on Mental Health: A Systematic Descriptive Review

**DOI:** 10.3389/fpsyt.2020.00074

**Published:** 2020-03-06

**Authors:** Paolo Cianconi, Sophia Betrò, Luigi Janiri

**Affiliations:** ^1^ Department of Neurosciences, Institute of Psychiatry, Catholic University, Rome, Italy; ^2^ Institute of Psychopathology, Rome, Italy; ^3^ Fondazione Policlinico Universitario A. Gemelli, IRCCS, Rome, Italy

**Keywords:** climate change, mental health, resilience, migration, vulnerability, climatic and economic turmoil, extreme events

## Abstract

**Background:**

Climate change is one of the great challenges of our time. The consequences of climate change on exposed biological subjects, as well as on vulnerable societies, are a concern for the entire scientific community. Rising temperatures, heat waves, floods, tornadoes, hurricanes, droughts, fires, loss of forest, and glaciers, along with disappearance of rivers and desertification, can directly and indirectly cause human pathologies that are physical and mental. However, there is a clear lack in psychiatric studies on mental disorders linked to climate change.

**Methods:**

Literature available on PubMed, EMBASE, and Cochrane library until end of June 2019 were reviewed. The total number of articles and association reports was 445. From these, 163 were selected. We looked for the association between classical psychiatric disorders such as anxiety schizophrenia, mood disorder and depression, suicide, aggressive behaviors, despair for the loss of usual landscape, and phenomena related to climate change and extreme weather. Review of literature was then divided into specific areas: the course of change in mental health, temperature, water, air pollution, drought, as well as the exposure of certain groups and critical psychological adaptations.

**Results:**

Climate change has an impact on a large part of the population, in different geographical areas and with different types of threats to public health. However, the delay in studies on climate change and mental health consequences is an important aspect. Lack of literature is perhaps due to the complexity and novelty of this issue. It has been shown that climate change acts on mental health with different timing. The phenomenology of the effects of climate change differs greatly—some mental disorders are common and others more specific in relation to atypical climatic conditions. Moreover, climate change also affects different population groups who are directly exposed and more vulnerable in their geographical conditions, as well as a lack of access to resources, information, and protection. Perhaps it is also worth underlining that in some papers the connection between climatic events and mental disorders was described through the introduction of new terms, coined only recently: ecoanxiety, ecoguilt, ecopsychology, ecological grief, solastalgia, biospheric concern, etc.

**Conclusions:**

The effects of climate change can be direct or indirect, short-term or long-term. Acute events can act through mechanisms similar to that of traumatic stress, leading to well-understood psychopathological patterns. In addition, the consequences of exposure to extreme or prolonged weather-related events can also be delayed, encompassing disorders such as posttraumatic stress, or even transmitted to later generations.

## Introduction

Since the 1970’s scientists have been trying to understand processes related to environmental factors that lead to climate change. Our climate is changing, with an unequivocal regional effect such as heath waves, floods, and droughts. Human activities have altered the atmospheric composition, producing a greenhouse effect leading to global warming. These activities produce a flux of complex variance with setbacks related also to mental health (1). Scientists argue that they still have to understand what kind of transformations can be expected depending on the temperature, how pervasive theses transformations will be in the various environments, when and what *points of no return* can be identified, which short and long-term consequences may be foreseen, and who are the most exposed biological subjects and most vulnerable societies. A part of climate change is due to the conditions of the planet that are independent of human activities (solar irradiance, autonomous activity of the planet such volcanic eruptions). Most studies are focus on the chain of events occurring in the biosphere primarily due to global warming. Global warming is in part attributable to the anthropogenic activity through the use of fossil fuels, deforestation, and pollution (1).

Global warming is likely to cause widespread emergencies in the future (2). These emergencies are real phenomena when extreme climatic events have an impact on local territories. These events are: extreme heat (increased global mean surface temperature, heat waves); climate change–related water disasters (CCRWDs) (sea level (3)—flooding, hurricanes, and coastal storms); droughts; wildfires; winter storms, extreme snow, and severe CAPE (convective available potential energy) thunderstorms (4) (supercells, derechos, and tornados).

When can we consider a climatic event as “extreme”? In science, the term “extreme” is used in several contexts. By definition, “extremes” are events that are rare or outside the normal range. A definition of extreme has been already deeply discussed in Seneviratne et al. report (5). Devastating natural weather phenomena are not exclusively caused by climate change. Some seasonal change or annual means temperature may be “extreme”. Therefore, extremes are understood within the context in which they take place. People and communities judge events as “extreme” by comparing them with personal experiences, when these events are unprecedented or divergent from previous phenomena (6). In the area affected by sudden and extreme climatic events, it is not rare to hear from the elderly that *“nothing like this has ever happened*”. However, focusing on a single climatic extreme and extraordinarily event does not allow to understand the bigger picture. Nonetheless, recently *extremes* are becoming more evident, even when a longer time arc is considered.

All weather events are affected by climate change. Higher global temperatures and differences in humidity compared to previous eras have been registered in recent years (6). There is some degree of uncertainty regarding climate change and the scientific community has not yet been able to fully link climate change to the increase of extreme weather events (7). However, many authors strongly believe extreme climatic events have important inﬂuence on ecosystems and societies. Shifts and trends of mean temperatures and precipitation have been directly correlated to the increases of hurricanes, droughts, heat waves, and heavy precipitation (8). Scholars have confidence that the anthropogenic influence has contributed to the increase of extreme events with disastrous outcomes on global scale (9).

The Katowice Climate Change Conference, held in Poland at the end of 2018, was the last conference concerning global change in an effort to commit each state to reduce emissions, trying to keep the global temperature change below 1.5°C. An increase of average global temperature over 1.5°C has been linked to a global rise in the frequency and intensity of extreme weather events (10, 11). Moreover, the greenhouse effect has already altered global climate dynamics (12). A vast amount of information supports that anthropogenic activity is responsible for extreme events, such as the heat waves in Europe and Russia (13), and the devastating floods in Pakistan (14, 15). Understanding how climate change relates to extreme events is a current scientific challenge. There are different explanatory experimental models (16). Each method must be able to explain the consequences of human activity and how they are linked to natural climatic variations.

Models on how global climate has evolved throughout the eras can be useful in order to give a context to current extreme events (17, 18). Such studies have increased over the past decades. Historical models and earth surface temperature readouts suggest that there is a strong connection between anthropogenic warming and the increased persistence of extreme weather (14). These approaches allow us to quantify the influence of historical global warming on the probability and the severity of individual events (19). All extreme climatic events are associated with large scale changes in the thermodynamic environments (12). For example, increases of mean temperature lead to heat waves (20); decreases in ground humidity and higher evaporation trends lead to higher incidence and severity of droughts (21) or changes in soil-moisture (22); high sea surface temperature and anomalies in humidity are linked to storms and the melting of Arctic ice fields (23).

Statistical methods link extremes to an observed climatic trend. This has proven to be challenging. The satellite era (1979–present) coincides with a rapid increase of Arctic surface temperatures. There is a high statistical confidence that links the anthropogenic action to the current minimum extension reached by Arctic glaciers, caused by the alteration of atmospheric circulation, atmospheric humidity, and thermodynamic factors in general (24). Authors strongly believe that some types of extreme event, most notably heat waves and precipitation extremes, will increase as an effect of the global warming (25). The frequency and the intensity of extreme climate events are unprecedented in global history (2). The debate is still open and, despite substantial progresses, achieving an accurate analysis of local events, evaluating all the involved thermodynamic processes, has proven to be difficult (24). Some studies already suggest that a more complex mechanisms may be involved in unprecedented extremes climate events (25).

However, our climate does not act as a linear system (26), but rather as a complex system (27), characterized by regime shifts, oscillations, and chaotic fluctuations in all timescales (26), so that predictions are impervious. Climate change will inevitably impose alterations in our lifestyles and consequences in terms of human losses and social readjustments. That said, it is not clear how many people on the planet will be affected by extreme phenomena caused by climate change, and to what extent and when these phenomena will compromise the quality of their life. People could be at risk for their survival, either directly (during, for example, an extreme event) or indirectly (reduction of food, famine, water scarcity, decrease in places to cultivate or hunt, displacement). In order to advise societies on how to cope and adapt to these phenomena, specialized international agencies have produced reports on climate change and the extreme events suggesting strategies and remedies (9, 28). Climatology, once a sub-field of physical geography, has now grown in relevance throughout the scientific community. Extreme events are the point of interaction between climate change and the human world (29).

Moreover, our ecosystem will face plant and animal extinction due to failure to adapt or migrate (30) leading to an increased risk of an “extinction domino effect” (31). Climate change can redistribute the strength of ecological interactions between predator and prey (32). Moreover, effects of global warming such as droughts and soil dry out (33) could have amplified effect especially in rural areas (34) such as difficulties in farming, starvation, forced migration (35); the consequent overcrowding of coastal and delta areas could also lead to physical illness by vector-borne disease. Global warming could pose a risk to ecosystems with decreased biodiversity, modifying fishing, and hunting activities (36). Famines can also be caused by abnormal insects’ populations and consequent increased use of pesticide or GMOs (37) with a change in biodiversity. Eighty percent of global population is affected by water and food insecurity due to climate change effects (38).

Environmental factors are becoming increasingly important in psychiatry due to the fact that they can induce congenital defects, impair neurodevelopment, even trigger endogenous mental disorders as well arouse psychosomatic and neurological disorders (39). Climate can produce strong phenomena with a disastrous impact among human societies. Disasters create a different kind of psychological and psychopathological distress compared to normal seasonal weather changes, as seen in tornados, floods, and droughts. Furthermore, other climatic events, usually overlooked in studies on mental health of exposed populations (e.g. ocean acidification, acid rain, *superfog* (40), glacier melting, biomass extinction), could also have a broader impact on mental health.

Psychiatry has only recently begun to deal with climate change, albeit specific literature concerning the climate events in relation to psychiatric disorders is still lacking and rather undefined (41–43).

## Materials and Methods

All papers available on PubMed, EMBASE, and Cochrane published from 1996 until June 2019 were reviewed. Searched terms included “PTSD” or “anxiety” or “depression” or “mental health” or “psychiatric disorder” or “psychosis” or “schizophrenia” or “suicide” or “mood disorder” combined with “climate change” or “extreme events” or “disaster events” or “surface air temperature” or “heath waves” or “rise temperature” or “floods” or “flooding” or “increased waters” or “hurricanes” or “tornado” or “drought” or “wildfires” or “vector borne disease” or “deglaciation” or “deforestation” or “river disappearance” or “increased of desert” or “extinction” or “solastalgia” or “ecoanxiety” or “ecomigration” or “resilience” or “adaptation”. The search included all languages and we focus on articles written in English or Italian. Studies related to both human and animal conditions were selected, including data available from government and non-governmental organizations, reports, and books.

Screening was made on the basis of abstract and title. Exclusion criteria are:

- Even in the event that direct effect on mental health was proven, articles on urbanization (n = 3), air and water pollution (n = 66), chemical pollution (n = 7), and ionizing radiation (n = 22) were excluded, insofar as they were not directly related to the focus of our study.
- Articles dealing exclusively with transmission of infectious diseases (n = 3) or physical-medical pathologies were excluded.

**Figure 1:**
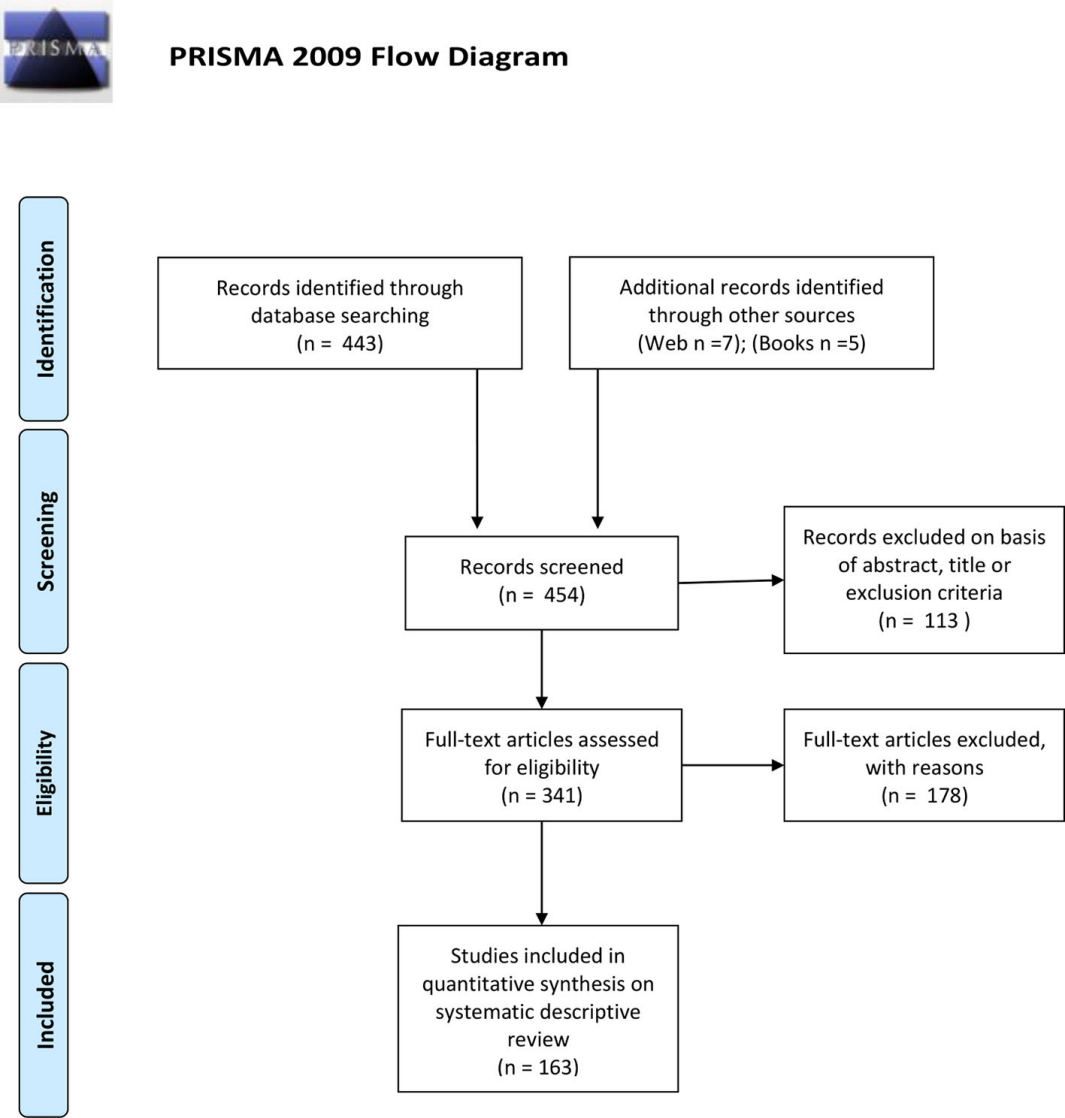
PRISMA flow diagram.

The following articles was eligible for analysis based on their specific topic. Exclusions (n = 178) were made in order to avoid redundancy of cited material (**Figure 1**).

- 100 articles on climate change in general—34 articles were selected;
- 18 articles on heat waves and temperature increase—six articles on heat waves and seven on temperature increase were selected;
- 31 articles on flooding and sea-level increase—20 were selected on flooding and 6 on sea-level increase;
- 20 articles on hurricanes—13 were included in this research;
- 10 articles on deforestation—seven were selected;
- 18 articles on drought—15 were selected;
- 25 articles on indigenous communities, vulnerability, and migration—18 were selected;
- 23 articles on economic impact—two were selected;
- 16 articles on wildfires—10 articles were selected;
- 84 articles on psychiatric disorders connected with global climate change—27 general articles were selected. Moreover, 28 association reports (WHO; IPCC; APA etc.) were studied—15 reports were selected.

In each category, only pivotal studies included in the range of the publication years were selected.

A total of 97 articles were analyzed, covering various extreme weather events and their effects on psychiatric illness. We examined 7 reports and 28 reviews; among these, 3 were about communities, 1 about migrations; one studied event and consequences in 157 countries, one summarized the interviews performed on 105,549 patients. We analyzed 8 articles with regression time studies (concerning 29 countries and data about surveys performed on 1,9 million citizens). Authors of 14 articles have given self-reported questionnaires or surveys (range of scrutinized results went from 30 to 381,916 people in internet questionnaires). Nine articles reported results from interviews, mainly about communities and children studies, ranging from 14 to 342 people interviewed. One of the selected articles is a population based retrospective study with 9 million data collected; one experimental study use statistic to analyze 344,957 *twitters*, another analyzed 600 million social media updates and reactions for a longitudinal and country scale evidence study. Four of the selected articles were case-studies with 17 people interviewed and 1,526 questionnaires completed. Excluding other minor selected categorized, other articles were longitudinal or cross-sectional studies.

## Results

### Course of Mental Health Changes Following Extreme Events

A vast body of works on mental health and climate change is now emerging (43, 44). Impacts on mental health can occur after or even before an extreme event (45). Mental health outcomes of climate change range from minimal stress and distress symptoms to clinical disorders, ranging from anxiety and sleep disturbances (46) to depression, post-traumatic stress, and suicidal thoughts (10, 47). Other consequences might include the effect on individuals and communities in their everyday life, perceptions, and experiences, having to cope, understand, and respond appropriately to climate change and its implications (10). A large number of people exposed to climate or weather-related natural disasters experience stress and serious mental health consequences. Some natural disasters are possibly going to be more frequent because of climate change. Notoriously, reactions to extreme events that involve life disruption, such as loss of life, resources, social support and social networks, or extensive relocation, are post-traumatic stress disorder (PTSD), depression, and general anxiety, increased substance use or misuse, and suicidal thoughts (48). Research has shown that peritraumatic experience is highly related to acute stress during and immediately after a disaster, which is expected to lead to the onset of PTSD (49). Later on, other consequences come out for survivors, such as reduced daily life activities and the loss of their “sense of place”. These conditions could have an impact and exacerbate mental health risks. News regarding climate change makes people uncertain and stressed, even depressed and with a sense of powerlessness. The concrete impact of those changes in life brings different types of psychopathological reaction to these events. Briefly, *acute* impacts refer to all the extreme events (e.g. floods, hurricanes, wildfires, etc.) that immediately expose undefended and helpless people to mental injuries. *Subacute* impacts involve intense emotions experienced by people who indirectly witness the effects of climate change, anxiety related to uncertainty about surviving of humans and other species and, finally, sense of being blocked, disorientation, and passivity. *Long-term* outcomes come in the form of large-scale social and community effects outbreaking into forms of violence, struggle over limited resources, displacement and forced migration (35, 50), post-disaster adjustment, and chronic environmental stress (51). In our study we considered the consequences for mental health for each single extreme event. We also considered mental health adaptation to environment changes.

#### Heat Waves

As a part of the Climate and Health Profile Report, both direct and indirect impact on health with regard to extreme heat and extreme precipitation were identified. Health risks caused by these factors has significantly risen in recent years (52). Heat weaves are spikes of high temperatures lasting some days that range outside the normal temperature for a specific season (20). This phenomenon is connected with climate change as they have increased in frequency and intensity. Moreover, the frequency and intensity of heat waves are considered extreme events linked to climate change, with a regional effect.

Physical health, mental health, human well-being, and heat waves appear to be specifically interconnected. Heat stress directly caused by heat waves has been associated with mood disorders, anxiety, and related consequences (53). People with mental illness were three times more likely to run the risk of death from a heat wave than those without mental illness (45). During pregnancy, especially in the second and third trimester, exposure to heat waves has been showed to be related to a lower average birth weight and increase of incidence of preterm birth. Effects during childhood and adulthood comprised reduced schooling and economic activity, other than behavioral and motor problems and reduced IQ (45). Some evidence seems to hint a different vulnerability between genders. The percentage of deaths were higher in women than men during the European heat wave. Negative outcomes of heat waves are also related to social factors. Women, young people, and people with low socioeconomic status have shown to be more vulnerable to anxiety and mood disorders related to disasters (54). Heat-related illnesses and waterborne diseases are also connected (52). It could also be noted that people are outside more during the summer, and this could increase the opportunity for conflicts. In hot temperatures, increase in discomfort leads to increase feelings of hostility and aggressive thoughts and possibly actions. Hotter cities were more violent than cooler cities. The increase in *heat-related violence* are greater in hot summers and showed increased rates in hotter years (55). Indeed, being exposed to extreme heat can lead to physical and psychological fatigue (53)—there is a clear association between warming temperatures and rising suicide rates, especially in an early summer ‘peak’ (56). Alcohol is likely to be involved in increasing aggression. Higher temperatures than usual, especially in June and July, were associated with an outbreak of aggressive crimes. The co-occurrence of hot summer days and weekends is therefore a perfect mix, resulting in a massive increase in shootings (57). Preventive measures should be taken against extreme temperatures to protect health, planning targeted public health monitoring, expanding the availability of air conditioning and providing countermeasures for what is known as the “urban heat island” effect (52).

### Water

Global warming will lead to CCRWDs such as an increase in both intensity and global number of tropical cyclones (58, 59), frequency and severity of hurricanes, and flooding (60).

#### Floods

Floods are an overflow of water, usually from the ocean, submerging land areas. Flooding is one of the most frequent types of major disaster, leading to 53,000 deaths in the past decade (61). CCRWDs generate flooding in urban areas on the coasts, like those in Asian deltas rivers (62, 63). These events could potentially have a negative effect on the health of vulnerable populations. Moreover, CCRWDs have a devastating impact on communities and the health of residents, for example by exposing people to toxins, precipitating population susceptibility, and creating crises for healthcare infrastructures (60). There are direct impacts in terms of morbidity and mortality related to water (such as drowning, electrocution, cardiovascular events, nonfatal injuries, and exacerbation of chronic illness); waterborne diseases (due to contamination of drinking water); infectious diseases (64); and psychiatric and mental health disorders. The principal effect after flooding seems to be located in the mental health area, leading especially to PTSD. A direct correlation between the intensity of the disaster and the severity of the mental health effects was noted (65). After the acute emergency phase, a number of members of the affected populations are subjected to some level of psychological distress and mental health problems (61). As studied all around the world (i.e. India and Bangladesh (61); Dakota USA (66); Italy (67); Venezuela (68) etc.) floods bring mourning, displacement, and psychosocial stress due to loss of lives and belongings, as a direct outcome of the disaster or of its consequences. All these are risk factors for PTSD, depression, and anxiety (69). With specific reference to flood victims, 20% had been diagnosed with depression, 28.3% with anxiety and 36% with PTSD (70). Consequences are still present well after the flood is over, due to the presence of mourning, economic problems, and behavioral problems in children (71, 72). Moreover, some cases show an increase in substance abuse, domestic violence, as the calamity exacerbates and precipitates previous existing people’s mental health problems (70, 73–75). Contradictory evidence was obtained regarding suicide following a flooding. In addiction there are aspects of vulnerability such as poverty (60, 76), living in makeshift housing solutions, and the lack of access to healthcare. Flooding disrupts infrastructure, causing problems for the standard systems of care, including mental health care that could assist and mitigate the psychological outcome for victims. Other susceptibility factors for mental illness related to these events include: women, the young or elderly age, having a disability, being part of an ethnic or linguistic minority, living in a household with a female head and having lower level of schooling. Among residents and relief workers, having limited resources or living in lower income countries are additional risk factors (77). The disaster can only exacerbate any existing barriers in the access of mental health care in the population (78). The focus of many articles is on PTSD occurring immediately after the disaster, when vulnerable people are more at risk and fragile (61). This part of the population can develop mental health problems and mental disorders in the short-, medium- or long-term (78). In addition, even families not residing nearby the affected area shown high levels of post-traumatic stress, due to the fact that they still bear the charge of the disruption of community cohesion (76). People who are affected by flooding could show remarkable resilience, however they still need organizations to support them, in order to recognize and cope with the distress, while also providing assistance to avoid any possible additional mental health problem or disorder arising from the situation. Psychosocial resilience, due to its personal and collective components, makes it fundamental to mitigate the distress of flooding together with the social and health risks caused by the event. Restoring social cohesion of communities and families immediately after calamities is crucial in order to reduce suffering and promote effective recovery (73). Community resilience also has a preventive effect, as it prepares the population for future events while also helping them to cope with the current situation (76).

### Air

Tornadoes, hurricanes, and storms have all increased in intensity, frequency, and duration over recent decades. Data on tornadoes and mental health issues came from the latest kind of this disasters, such as hurricane Katrina in Florida and Louisiana in 2005 and Sandy in 2012 in Cuba, Jamaica, and Haiti (79–81). There is still uncertainty regarding how much humanity is at fault for this increase (82). Based on information provided by the United Nations Development Program, nations like the United States, Japan, Australia, and New Zealand and other twenty-nine developing nations have been greatly exposed to hurricanes, cyclones, and typhoons (83). The damage suffered by health care infrastructure and the interruption of public health service due to hurricanes leads to an increase in serious illness, injuries, disability, and death (84). As in extreme events, there are health issues that emerged or worsened after hurricanes due to psychological stress: increase in rates of cardiovascular diseases (85), prenatal maternal stress and depression, infants more likely to experience anxiety, fear and sadness, and less responsive to pleasant stimuli (86), lack of insurance possibly increasing chronic illnesses with no access to medical care during a disaster (87, 88), population exposures to contaminated floodwaters (88). Many people experienced PTSD, stress, depression, anxiety (87), and suicide (79). Consequences of material damages lead to substance abuse (45, 89). The incidence of PTSD, that has been studied most extensively, was consistently associated with several factors. Severity of exposure and previous mental health problems has shown to be stable predictors of development of distress (84). Other vulnerability factors are represented by: age, women, low education level, low socioeconomic status, being unemployed or disabled before hurricane disaster, and being single (54). People living in an affected area showed high levels of suicide and suicidal ideation, one in six people developed PTSD, and half of them developed an anxiety or mood disorder, including depression (45). Additional consequences are the loss of social support, job insecurity, and loss of belongings, as well as disruption of medical health system, displacement, and relocation are related to the onset of psychological distress. Mental health disorders are often seen even one year after the disaster or event (90). A strategy for coping immediately after a hurricane is a successful evacuation of vulnerable areas by reducing the number of victims. Displacement to shelters often results in separation from social support networks and creates a disruption in normal psychological processes, particularly familiarity, attachment and identity, and decrease in perceived social support in the months following the hurricane, which in turn has been shown to be associated with increased symptoms of general psychological distress. Being moved from one shelter to another is traumatic, compounded by the limited amount of healthcare services (91).

### Drought

Historically, a natural drought lasts about a decade. Due to climate change, there will be droughts lasting around three decades, also known as “megadroughts”. From a current historical frequency of 12%, these events may increase up to 60% (92) due to possible changes in future anthropogenic greenhouse gas emissions and atmospheric concentrations measured in CO2-equivalents (93). A combination of high temperatures and low precipitation increases the frequency of drought over the world (94). A temperature fluctuation is correlated with agricultural loss by affecting crop productivity and yields. This loss is linked to a decrease on economic growth leading to a long-term economic disadvantage and promoting political instability and conflicts (95). Farmers all over the world are more vulnerable to environment-induced mental health risks carried by drought. Long-term droughts and erratic rain fall have been associated with deterioration of economic conditions, reduced social functioning, and psychological distance to perception of negative climatic conditions (96). The regulation and adjustment of emotion is disrupted by depression, demoralization, fatalism, passively resigning to fate, especially in women and adolescents or people with lower socioeconomic status, showing feelings of distress and helplessness (52, 53). Drought has been often connected to suicide (97–101), especially in older people (102). In semi-arid and peri-urban areas, adaptive capacity is necessary to cope with an increased temperature and a reduced rainfall. In this case, vulnerability is seen as the degree to which people are susceptible by events that disrupt their lives—events that are beyond their control. Resilient systems cope with extreme events in order to create a response that maintains essential function, identity, and structure, and the capacity for adaptation. Consequently, local communities strongly perceive the impact of climate change. Negative events stimulate feelings of alertness, constant monitoring of current and future events, mental distress, anxiety, depression, and suicide (103), as well as prolonged emotional stress, inevitably provoking high job insecurity (51) and other psychological issues (104).

Drought and migration are related through an assessment of crop yields (105). The landscape changes with periodical drought impact for example on the Turkana’s ability to gain access food, water, health, and security. These indigenous people are farmers that have grown dramatically in number in the last decades with a majority living below the poverty line. Prolonged and more frequent droughts and unpredictable rainy seasons have exacerbated the difficult access to potable water or food. Consequently, changes in water availability, temperature or other environmental variables can have a truly devastating impact on their daily life. Many extreme weather events and famine lead to displacement of entire communities and forced migration, within and outside national borders, with onset on conflicts over natural resources (106). In northern Ghana’s savanna (an arid zone with severe droughts), climate change exposes farmers to adverse climatic conditions that include low rainfall, forest fires, soil erosion, loss of soil fertility, poor harvest, and destruction of property and livestock. In this region, farmers with small plots are at a higher risk of suffering acute, sub-acute, and long-term problems caused by extreme climate change (51).

### Wildfires

The term “wildfire” refers to large-scale fires, generally occurring in forests and jungles. These phenomena have involved Siberia, Central Africa, and the Amazon in the present times. The areas affected by the wildfire may be sparsely populated or nearby the city boundaries. The greatest concerns are those related to the climate. The effects on the ecosystem are devastating, as a forest’s carbon dioxide storage capacity is forever lost (107). Once burnt, forest tend to become savannah, scarcely covered by deciduous trees or cultivation. Vast decline of forests will have reverberations for the world’s climate, as anticipated by many government administrations and institutions all over the world. A “business as usual” scenario will lead to a rise in temperature of about 4 degrees Celsius by 2100 and plants will have to find new strategies to survive.

In the past, a more local phenomenon has been described as bush-fire. These involved urban areas in proximity with bushlands or forests and could affect residential neighborhoods, suburbs or slums in different ways, albeit devastating for farmers and people in the area. Firestorms generated by bush-fires led to destruction and evacuation of residents. Wildfires have a heterogeneous impact on property damage, physical injuries, and mental health (108). During the years following the disaster, an increase of mental health issues was observed, such as general mental health problems, post traumatic disorders (109), psychosomatic illness, and alcohol abuse (110). Effects can be delayed in onset and can persist over at least several years. Proximal populations, not directly affected by the bush-fires, can also be involved (111). Studies performed in areas hit by Australian bush-fires observed that a year after the events 42% of the population exposed was classified as potential psychiatric cases (109). Californian wildfires also offer a rather dramatic picture, with 33% showing symptoms of major depression and 24% showing symptoms of PTSD. A similar effect was observed in Greek wildfires (112), with an increased somatization symptoms, depression, anxiety, hostility, and paranoia (113). Post-disaster mental health issues observed were PTSD, physiological hyperarousal, chronic dissociation, sadness and depression, detachment (114) disorganized thinking and behavior, numbing or avoidance, poor concentration, and behavioral problems. In the youth population, connections have been found between the young age and the experience of personal life threat (115). Children were also affected by bush-fires, showing posttraumatic phenomena such as anxiety disorders and panic attacks, problems sleeping, acute stress disorder, compulsively repetitive play, flashbacks, and psychotic disorders (116).

Wildfires could become quite frequent with climate change. Addressing such phenomena on a global scale will prove challenging. It is also difficult to predict how populations will react once the anthropogenic role of this type of event takes hold.

### Long-Term Environment Changes and Critical Psychological Adaptations

Climate change leads to extreme events that bring an immediate and direct impact on the population in terms of mental health. However, changes also occur slowly, as in the case of temperature increase. Climate change will also modify the representation of territories as historically and traditionally known and lived by the populations. This loss of spatial and cultural parameters is not favorable to the people’s life in terms of changes of lifestyle that they might undergo. People may also become less familiar with places and usual products (resources) of the environment. Landscape changes are brought about by deforestation, deglaciation, river disappearance, desertification, water shortage, increase of infectious diseases, and biomass extinction. On its own, desertification (117) may cause resource-based conflicts (106).

#### Increase of Average Land Surface Air Temperature

The earth could become very hot due to the anthropogenic effect of greenhouse gases that alters the dynamics of CO2. Moreover, recent studies have highlighted how climate change and global warming are nonlinear trends (118).

The increase in environmental temperature can notoriously compromise the functioning of the central nervous system, similar to insolation and heat stroke. The correct temperature for proper functioning is around 22°C (119). Outside temperature can affect the risk of onset or continuous mental disorders in different ways. For example, temperature stress can influence psycho-physiological functions by directly affecting bio-chemicals levels, (e.g. altering the production of serotonin and dopamine) (120) or by disrupting the homeostasis of thermoregulation (121, 122). Additionally, direct heat could result in sleep disturbance, exhaustion, and heat stress associated with suicide (56, 123). Moreover, the correlation between mental illness and increase of temperature depends on latitude and factors that go beyond geography, such as cultural, political, and socio-behavioral factors (120). In the tropics, temperature fluctuations show a clear connection to negative agricultural, economic, and political situations. This is clearly seen in poor countries, which are more vulnerable to weather and climate fluctuation than wealthier countries (95). As described above, a single disaster, like a hurricane or flooding, has profound effects on mental health, in direct and acute manners. Furthermore, heat waves and extremely high temperatures expose the population to the possible onset of mental disorders. Sensitivity of mental disorders to temperature should not be underestimated when compared to other heat-related physical diseases that are commonly studied. Naturally, the levels of exposure to high temperatures can vary, affecting populations in different geographical areas, thus leading to greater challenges in the study of risks (120). Studies show higher risks of mental-disorder correlated to warmer temperatures, specifically mania in the elderly, as well as a positive association with transient mental disorders and episodic mood disorders and an increase in hospital admissions for mental illness within a few days after warmer temperatures. When temperature rose over a threshold of about 20°C, there is an increase in drug-related mental disorders. Furthermore, there is also a correlation with increased mortality and morbidity risks among people with mental and behavioral disorders (120). Increases in hospital emergency room visits are also shown for many of mental illnesses such as mood disorders (124), substance abuse, behavior disorders, neurotic disorders, and schizophrenia and schizotypal disorders. People are more affected by high temperature especially if they have schizophrenia, schizotypal disorders, and mood disorders (121). During the increase of temperature, there is a risk of mental states of aggression resulting in violence and self-harm, inflicted injury/homicide, and self-injury/suicide (122). Many studies found no significant associations with cold temperatures (51, 120).

#### Increase in Sea Level

Global sea level is projected to rise between 30 to 121 centimeters by 2100, due to the influx of water from melting glaciers and the expansion of seawater as it warms (125). There are many factors that contribute to the increase of water (melting glaciers specifically in Greenland and Antarctic as well as worldwide), as well as changes in rainfall patterns and increasing frequency of severe weather such as flooding (126–129). Countries with low-lying areas, small islands like those in the Indian or Pacific Oceans, are concerned that their land areas might decrease due to flooding and coastal erosion. Consequently, many people could be forced to migrate to other countries leading a persistent worry and thoughts of relocation (128). Specific fears of encirclement or siege by the sea would replace the population’s normal relationship with the sea or ocean (130).

#### Deforestation

Deforestation occurs due to the loss of plant biomass caused by climatic events and the direct action of mankind, driven by agriculture, animal grazing, and mining (131). There has been an enormous loss of forests due to human activity. News regarding such events have a stressogenic impact on western populations, due to the increased ecological awareness. People believe that an important world heritage has been damaged and lost. This feeling is now known as a *biospheric concern*. On the other hand, for indigenous populations, deforestation has a deeper impact, leading to profound maladaptive disorders and depression. In general, people believe that forests are a source of health and protection from various types of stress (132).

Urban green areas help maintain low temperatures in the city during the summer months, improve air quality, and reduce people’s stress level (133). An ever-increasing number of studies shown that living in green urban spaces leads to health benefits, including better physical and mental health and a longer life expectancy (134). Studies suggest that the positive influence of nature on health can be observe especially between vulnerable groups such as the elderly, those in rehabilitation for mental disorders, and individuals in crisis rehabilitation (135). In the older population, contact with parks and green areas has been linked with slower cognitive decline. Moreover, children benefit from living in greener urban areas, such as better spatial working memory, improved attentional control and capacity, and higher academic achievement, particularly in mathematics (136), as well as improved behavior and emotional development, and positive structural changes in the brain (134). Moreover, green areas and parks in childhood have a preventive effect on the risk of developing various mental disorders later in life. More greenery in urban areas creates higher social cohesion and increases people’s physical activity level, therefore improving children’s cognitive development (137). In general, subjects living more urban green areas have a better quality of life. Unfortunately, those people who could get more benefits from a change of exposure often do not have sufficient resources or capacity to move to more healthy environments (138).

*Landscape modification:* landscape modification can induce individuals to develop a profound sense of loss of connection and detachment from the environment they know. Solastalgia is a term that describes this type of loss, especially when a person finds it difficult to adapt to environmental changes, possibly creating risks for mental health (53). Solastalgia describes a complex phenomenon that can have an impact on psychological levels, similar to that experienced by people who are forced to migrate (139). *Biospheric concern* refers to a type of stress that people feel when they see vulnerable nature such as plants or animals and the environment (140). Daily life can undergo variation due to climate change and when people lose their autonomy and control, they could experience a deep psychological change. For example, the loss of employment related to environmental changes can lead to a loss of individual identity. Immediately after a disaster, damages on social or community sources, or lack on food and medical services, could results in many acute consequences for the psychological well-being (139). In contrast, slow change of the environment due to climate change, like changes in usual weather or rising sea levels, will cause acute and chronic psychopathologic trauma and shock, PTSD, depression, anxiety, suicide, substance or alcohol abuse, aggressiveness and violence, difficulties in social and interpersonal relationships, loss of personally important places, alteration of social ties, loss of autonomy, and control, as well as personal and professional identity, leading to the emergence of feelings of helplessness and fear, solastalgia, and eco-anxiety (45).

#### Economic Impact

After ecological and environmental changes in a country, economic crises can occur and lead to an increase in suicide rates and other mental and behavioral disturbances especially working men (141). This type of stress, when associated with low socioeconomic status, paired with limited access to resources and reduced health, can lead to a diminished ability to cope. Economic difficulties that last over time or in conjunction with other factors, lead to a physical, cognitive, psychological, and social malfunction with a decrease in well-being and health (142). Both economic and climatic variables are strongly correlated with suicide, with 62.4% of male and 41.7% of female suicide rate variability across the continent (141).

### Exposure Groups

Certain people are indeed more vulnerable to the potential impacts of climate change on mental health. There are communities that are more vulnerable to such events. This impact implies psychological effects (45) especially in vulnerable groups like children, the elderly, the chronically ill, people with mobility impairments, pregnant and postpartum women, people with mental illness, and those with lower socioeconomic status. Consequently, climate change has worsened global economic inequality (143). The more temperature shifts from optimum levels, the more there will be groups that cannot cope. A striking example is the traditional native populations. Different studies have considered the effect of climate change on native communities (such as first nations and aborigines), highlighting aspects of vulnerability and resilience (144, 145). Among these populations, the elderly is a clear example of difficulty in re-adaptation. These minorities and at-risk groups, such as the Inuit communities, first nations, and aborigines (146), are experiencing rapid change in climatic conditions (132, 147, 148). In the Canadian arctic, the Inuit refer to having a protective factor for their mental health and well-being in “being on the land”. Melting ice and change in weather conditions are strongly linked to the impairment of these protective factors due to a decrease in access to land with some of the highest rates of youth suicide that have been documented among Inuit youth (149).

Climate change is a social determinant for mental health (150). Strong impact has been noted on refugees and migrants (151), ethnic minorities, the homeless or vulnerable populations such as the poor in countries like India, China, and Brazil (152). Women, those with low socioeconomic status, living in poverty, with scarce economic and social resources, reduction of social support, and mental health problems existing before the events, along with traumatic experiences (death or life risk, serious injury or sexual assault) and stressors, represent vulnerable groups that tend to develop new mental disorders or see their previous problems worsen (49). After climate disasters, children typically show more severe disturbances than adults, with more severity and prevalence with regard to the onset of PTSD and depression. (71).

Residential populations in changing territories are subjected to new environmental conditions. For these people, this violation of the usual context is experienced with passivity and a sense of powerlessness. Many studies show that when people experience feelings of loss, helplessness, and frustration caused by their inability to cope with climate change, a term today coined as *ecoanxiety* (45, 153). People may also experience feelings of uncertainty and anticipation of the unknown regarding climate change. This leads to a psychological distance, a perception of distance, when a climatic event occurs as near or far, at a temporal, spatial, and s ocial level (51). In response to growing ecoanxiety and various type of biospheric concern, psychotherapists are pioneering a new field of treatment, termed “ecopsychology”. It is important for doctors to teach patients to accept their own powerlessness. For example, when it starts raining, some patients have episodes of anxiety because they think of the past flooding with fear of losing the house again due to the flood. “Ecological grief” is a recorded grief and anxiety spread among the native Inuit to describe what they have seen (154).

These new words are emerging from recent observations on the impact and power that climate change has on mental health. It will certainly take time and further studies in order to identify these new diseases and disorders. The *DSM-5* and *ICD-10* offer no specific references to mental disorders related to climate change. The chapter “*Other conditions that may be a focus of clinical attention*” in the *DSM-5* contains the section “*Economic Problems*” where the following conditions are listed: lack of adequate food or safe drinking water, extreme poverty, low income, insufficient social insurance, or welfare support. In the section “*Problems Related to other Psychosocial, Personal and Environmental Circumstances*” the following conditions are listed: exposure to disaster, war, or other hostilities (155).

Certain groups and communities are now beginning to experience disruptions with regard to social, economic, and environmental determinants. When exposed to climate change, a population experiences constant uncertainty, anxiety, loss, disruption, displacement, and fear even before a disaster has even occurred. Climate change negatively impact on mental health and wellbeing with unequal distribution within and among communities (156). After a natural disaster has occurred, damage and efforts to repair it have increased the disparity of wealth between races (this has been shown in United States). There has been a clear-cut increase with regard to inequality in countries that are frequently hit by extreme events. When certain areas receive more redevelopment aid, racial inequality is going to be amplified (157). When farmers in various parts of the world perceive the psychological pressure of climate change, they are motivated to engage in different strategies in order to adapt to climate change (51).

## Discussion

### Summary of Main Findings

There is a strong link between natural disasters and mental disorders. In the future, climate change will bring about an increasing frequency of extreme weather. We know that weather changes may induce psychopathological phenomena such as seasonal affective disorders to weather sensitivity and meteoropathic conditions. Specific symptom patterns, below the pathological threshold, may be devised in reaction to various atmospheric changes and perturbations: temperature, humidity, rain, barometric pressure, brightness, rate of air flow, air ionization, thunderstorms, and sudden shifts of some of these factors (158). What is also seen as a temperamental trait has been called “meteorosensitivity”. Living organisms may be biologically more prone to suffer the effect of atmospheric events on mind and body. On the other hand, meteoropathic subjects are those individuals who develop a specific illness or the worsening of an existing disease as a consequence of climatic changes. Psycho-physical symptoms include: mood disturbances, irritability, anxiety, mental and physical weakness, hypertension, headache, hyperalgesia and pains, and autonomic symptoms (159). Moreover, air pollution can induce neural instability (158). Scarce rain and low average temperature have been found to lead to psychiatric visits in emergency departments (160). Hippocrates himself wrote: “Whoever wishes to investigate medicine properly, should proceed a so: in the first place to consider the seasons of the year … then the winds, the hot and the old … we must also consider the qualities of the water …” (161). Weather can impact everyday activity and changes in the behavior result from physical characteristics of the environment. With global climate change, these psychopathological phenomena due to sensitivity to normal weather conditions can today be studied within a wider dimension.

Climate change can lead to extreme weather, which include large storms, flooding, droughts, and heat waves, and it has effects not only on physical health (e.g. degraded air quality) through the spread of diseases and the reemergence of existing diseases, but also on mental health. Mental health consequences of natural disasters cover a wide range of disorders.

The connection between climate change and its consequences on mental health is far from reaching a clear conclusion. The complexity of current studies highlights this challenge. This difficulty is largely due to the heterogeneity in what to measure and how to measure the impact of climate change. Attempts to discover the underlying mechanisms of adaptation, as well as the definition of deviations from normality in extreme climate events, and finally attempts to define direct cause-effect relationships are all challenging tasks. Socio-behavioral factors, culture, information, and preparedness all play a relevant role in peri-traumatic experience, determining collective resilience or psychological disruption and exhaustion. Studies that empirically established connections between climate change and mental health consequences are now coming forth in literature. Impact of climate change on mental health can occur either *directly with immediate effect* (heat waves), or *indirectly in the short term* during extreme events (floods, tornadoes, hurricanes), or *indirectly in the long term* (changes in the territory such as prolonged droughts, increase in the sea levels, deforestation, forced migration). All these events affect the mental health of a population, with the appearance of psychiatric conditions such as PTSD, mood disorders such as depression, anxiety, increased suicide rate and substance use, as well as increased aggressive behavior. Climate change will also exert the greatest impact on groups of vulnerable populations that therefore have an increased probability of developing psychopathologies: women, the elderly, children, people with previous psychiatric illnesses who can consequently worsen their mental condition, and people with low income or poor social network, as well as indigenous and native communities. Extreme weather events seem to have the power to also destroy social ties (162). Vulnerable communities are those located in exposed regions (e.g. coastal regions, where windstorm or extreme heat can occur).

Climate change will produce profound changes in the environment and alter lifestyles, while also generating environmentally-motivated migration (random asylum seekers and climatic refugees). These groups of people, forced to migrate, already have their own psychological vulnerabilities (162). They may find it difficult even to identify the appropriate emotional control for specific climate changes. Moreover, extreme events produce different types of psychopathological reactions over time, as there are acute, sub-acute, and long-term impacts on mental health. Mental adaptation and certain behavioral patterns will develop following the chronology of events: in the pre-alert phase, during the disaster and after the event (163). Long-term consequences are difficult to define. Consequences of climate change, such as economic and social difficulty, contribute not only to the increase in the incidence of mental illnesses in the affected population, but also in the subsequent generations. Literature analyzes single types of climatic events, since certain consequent disorders are specific while others generally occur in different extreme events (162). Being able to understand what this change entails makes it possible to program early interventions and actions for a population’s mental health.

### Limitations

Studies on the consequences that climate change has on mental health are still at their very beginning. In the future, it would be useful to further investigate the correlation between psychiatric diseases and extreme events. We did not find any study on how people react to the changes in landscape such as deglaciation, disappearance of rivers, desertification, fires, and water shortages. A greater understanding of the characteristics of acute, sub-acute, and long-term consequences is an also desirable goal. Furthermore, we believe that future research in climate change and mental health will include multi-disciplinary studies. Scholars should focus on how different vulnerable groups can be affected by natural disasters and climate change, as well as how to make use of the available protective measure and healthcare resources. A limitation of the present descriptive review is the lack of a meta-analysis as a methodological completion of the systematic review. This could be useful in the upcoming research in order to establish specific causal associations between climate change and mental health consequences (symptoms and disorders).

### Conclusions

Based on the studies and literature reviewed in this paper, there appears to be strong evidence of the influence that the climate change exerts on mental health.

This study examined the effects of global climate change on the general population, as well as at-risk groups and vulnerable communities. We chose to focus on extreme events, such as those produced by temperature increase, heat waves, floods, drought, tornadoes, hurricanes, and wildfire. Consequences have been described in terms of distress symptoms, suicide rates, and clinical disorders (depression, anxiety, sleep disturbances, PTSD, etc.). Even though some of these events may occur in a slower and less acute manner (e.g. temperature increase or droughts), most of these events are rapid in their onset and manifest themselves in the form of disasters, the reactions to which often see PTSD as a prototypical model. On the other hand, we could support that people who are more sensitive to weather and atmospheric phenomena may be more affected by gradually occurring global climate changes and their consequences, such as global warming, rising sea levels, landscape changes, and loss of familiar environmental landmarks.

Moreover, the disappearance of animal and plant species may bring about feelings of hopelessness and depression. When a person’s feelings about their environment are considered, it should be clear that we are moving toward a cultural and contextual dimension. The wound inflicted to this symbolic domain causes more complex psychopathological consequences, such as identity disorders (164) or long-term personality changes (119), as seen in trauma related to extreme weather events and loss of familiar landscape, or dissociative syndromes (165) as seen in trauma related to extreme events or in migratory syndromes. Lastly, we also need to learn how meteoropathy and weather sensitivity, paired with environmental and climate changes, deeply influence the psychosomatic sphere of mankind, activating mechanisms of somatization and conversion, and inducing somatic disorders and physical illnesses or worsening previously existing distress at the body level. These psychiatric disorders, in quality and quantity, are linked to the type of evolution of post-modern societies. In short, all of these issues need to be more extensively studied and clinical experience should be gained in order to support our provisional conclusions. The challenge of climate change will be protracted in the upcoming years. Therefore, this branch of “ecopsychiatry” will surely be supported by new data sets and further studies.

## Author Contributions

PC, SB, and LJ all contributed to the brainstorming, writing, and critical review of this manuscript. LJ edited the manuscript.

## Funding

Funding for this study was provided by Fondazione Policlinico Gemelli - Institute of Psychiatry, Catholic University, Rome, Italy. The funders had no role in this study design, data collection and analysis, decision to publish, or preparation of the manuscript.

## Conflict of Interest

The authors declare that the research was conducted in the absence of any commercial or financial relationships that could be construed as a potential conflict of interest.
